# Increased risk of SARS-CoV-2 reinfection associated with emergence of Omicron in South Africa

**DOI:** 10.1126/science.abn4947

**Published:** 2022-03-15

**Authors:** Juliet R. C. Pulliam, Cari van Schalkwyk, Nevashan Govender, Anne von Gottberg, Cheryl Cohen, Michelle J. Groome, Jonathan Dushoff, Koleka Mlisana, Harry Moultrie

**Affiliations:** ^1^ South African DSI-NRF Centre of Excellence in Epidemiological Modelling and Analysis (SACEMA), Stellenbosch University, Stellenbosch, South Africa.; ^2^ National Institute for Communicable Diseases, Division of the National Health Laboratory Service, Johannesburg, South Africa.; ^3^ School of Pathology, Faculty of Health Sciences, University of the Witwatersrand, Johannesburg, South Africa.; ^4^ School of Public Health, Faculty of Health Sciences, University of the Witwatersrand, Johannesburg, South Africa.; ^5^ McMaster University, Hamilton, Ontario, Canada.; ^6^ National Health Laboratory Service, Johannesburg, South Africa.; ^7^ School of Laboratory Medicine and Medical Sciences, University of KwaZulu-Natal, Durban, South Africa.; ^8^ Centre for the AIDS Programme of Research in South Africa (CAPRISA), Durban, South Africa.

## Abstract

Here, we provide two methods for monitoring reinfection trends in routine surveillance data to identify signatures of changes in reinfection risk and apply these approaches to data from South Africa’s SARS-CoV-2 epidemic to date. While we found no evidence of increased reinfection risk associated with circulation of Beta (B.1.351) or Delta (B.1.617.2) variants, we find clear, population-level evidence to suggest immune evasion by the Omicron (B.1.1.529) variant in previously infected individuals in South Africa. Reinfections occurring between 01 November 2021 and 31 January 2022 were detected in individuals infected in all three previous waves, and there has been an increase in the risk of having a third infection since mid-November 2021.

As of 31 January 2022, South Africa had more than 3.6 million cumulative laboratory-confirmed cases of SARS-CoV-2, concentrated in four waves of infection (Fig. 1). The first case was detected in early March 2020 and was followed by a wave that peaked in July 2020 and ended in September. The second wave, which peaked in January 2021 and ended in February, was driven by the Beta (B.1.351 / 501Y.V2 / 20H) variant, which was first detected in South Africa in October 2020 (*1*). The third wave, which peaked in July and ended in September 2021, was dominated by the Delta (B.1.617.2 / 478K.V1 / 21A) variant (*2*). In late November 2021, the Omicron (B.1.1.529 / 21K) variant was detected in Gauteng Province, the smallest yet most populous province, and associated with rapidly increasing case numbers (*3*). The estimated effective reproduction number in Gauteng based on PCR-confirmed cases was 2.3 as of 18 November 2021, which was as high as had been seen at any point during the prior three waves, and peaked above 3 in late November (*4*, *5*). The proportion of positive PCR tests with S-gene target failure (SGTF), a marker of the BA.1 sublineage of the Omicron variant, subsequently increased across all provinces (*6*).

**Fig. 1.**
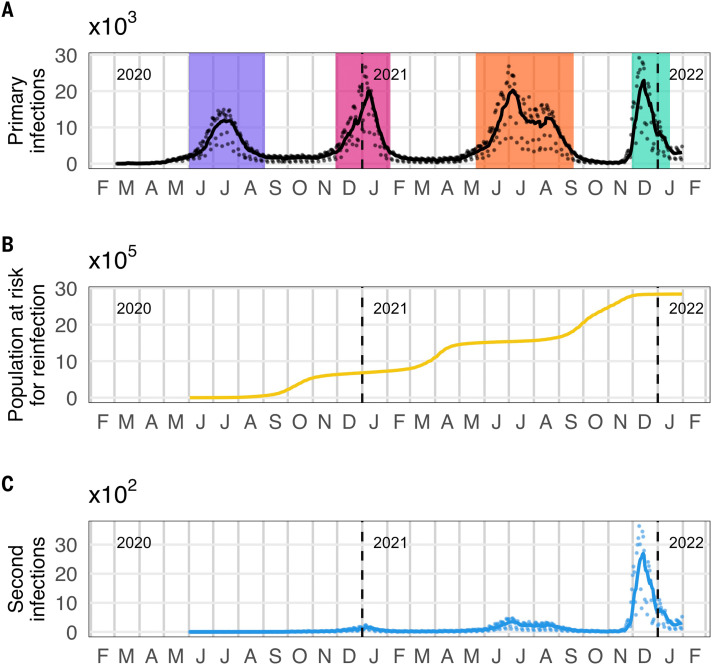
Daily numbers of detected primary infections, individuals eligible to be considered for reinfection, and suspected reinfections in South Africa. (**A**) Time series of detected primary infections. Black line indicates 7-day moving average; black points are daily values. Colored bands represent wave periods, defined as the period for which the 7-day moving average of cases (detected infections and reinfections) was at least 15% of the corresponding wave peak (purple = wave 1, pink = wave 2, orange = wave 3, turquoise = wave 4). (**B**) Population at risk for reinfection (individuals who tested positive at least 90 days ago and have not yet had a suspected reinfection). (**C**) Time series of suspected second infections. Blue line indicates 7-day moving average; blue points are daily values.

Following emergence of three variants of concern in South Africa, a key question remained in late 2021 of whether there was epidemiologic evidence of increased risk of SARS-CoV-2 reinfection with these variants (i.e., immune escape from natural infection). Laboratory-based studies suggest that convalescent serum has a reduced neutralizing effect on the Beta, Delta, and Omicron variants compared to wild type virus in vitro (*7*–*12*); however, this finding does not necessarily translate into immune evasion at the population level.

To examine whether reinfection risk has changed through time, it is essential to account for potential confounding factors affecting the incidence of reinfection: namely, the changing force of infection experienced by all individuals in the population and the growing number of individuals eligible for reinfection through time. These factors are tightly linked to the timing of epidemic waves. We examine reinfection trends in South Africa using two approaches that account for these factors to address the question of whether circulation of variants of concern has been associated with increased reinfection risk, as would be expected if their emergence was driven or facilitated by immune evasion.

## Identification of and characterization of reinfections

We define a suspected reinfection as a positive SARS-CoV-2 test in an individual with at least one previous positive test and whose most recent positive test occurred at least 90 days earlier. Based on routinely collected line list data maintained by the National Institute for Communicable Diseases (NICD) with specimen receipt dates between 04 March 2020 and 31 January 2022, we identified 105,323 individuals with at least two suspected infections, 1,778 individuals with at least three suspected infections, and 18 individuals with four suspected infections.

## Time between successive positive tests

**Fig. 2.**
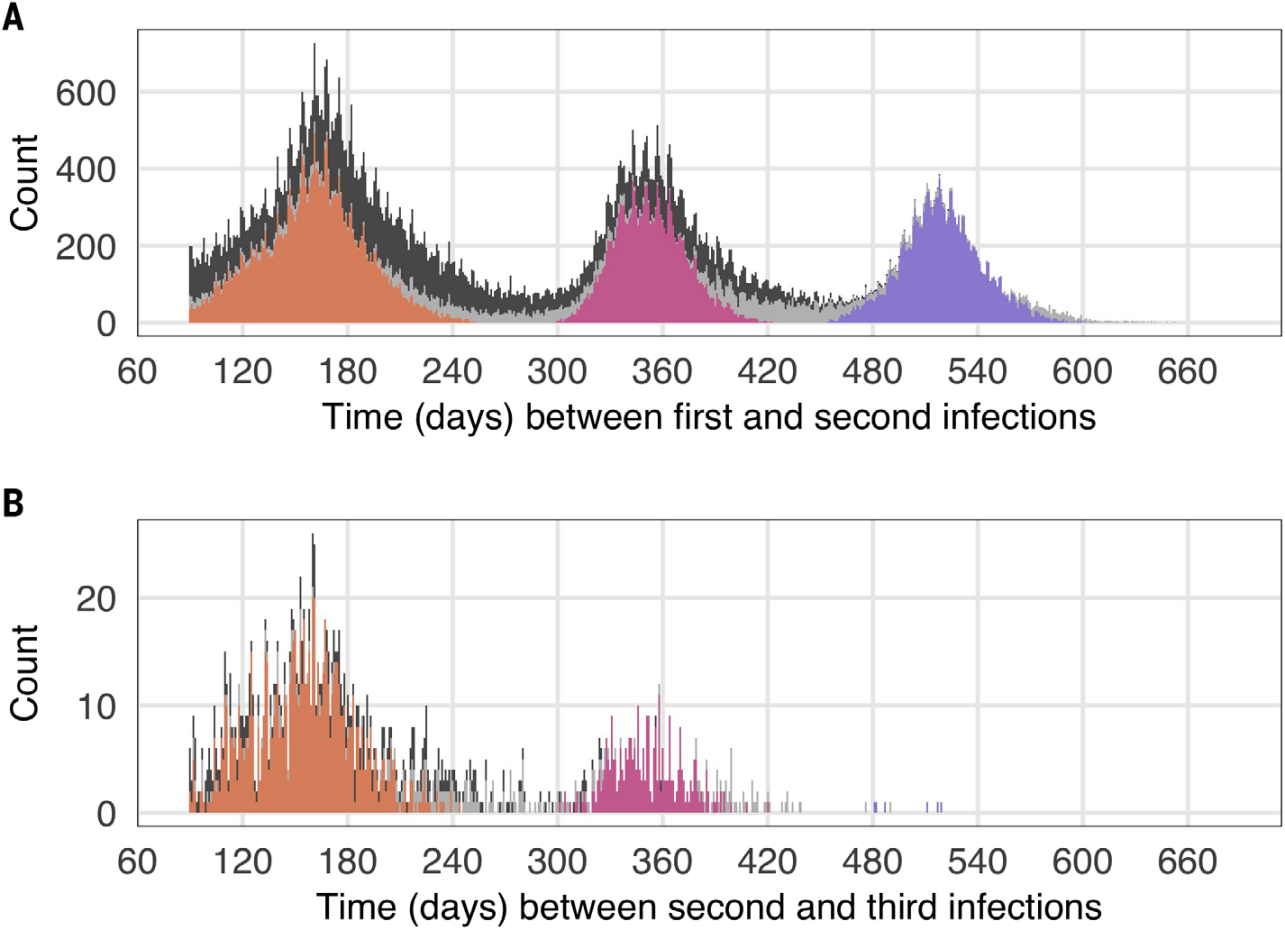
Time between consecutive infections, based on the time between successive positive tests. Note that the time since the previous positive test must be at least 90 days to be considered a reinfection. (**A**) Time in days between the last positive test of the putative first infection and the first positive test of the suspected second infection. (**B**) Time in days between the last positive test of the putative second infection and the first positive tests of the suspected third infection. Colors represent suspected reinfections diagnosed on or after 01 November 2021. In both panels, bars for these individuals are colored by the wave during which the previous infection occurred (purple = wave 1, pink = wave 2, orange = wave 3, light grey = inter-wave).

The distribution of times between successive positive tests for individuals’ first and second infections has peaks near 170, 350, and 520 days (Fig. 2A). The shape of the distribution is strongly influenced by the timing of South Africa’s epidemic waves, which have been spaced roughly six months apart. The first peak corresponds mainly to individuals whose primary infection and second infection occurred in consecutive waves (e.g., initially infected in wave 1 and reinfected in wave 2, initially infected in wave 2 and reinfected in wave 3, or initially infected in wave 3 and reinfected in wave 4), while the second peak corresponds mainly to individuals initially infected in wave 1 and reinfected in wave 3 or initially infected in wave 2 and reinfected in wave 4. The third peak corresponds to individuals initially infected in wave 1 and reinfected in wave 4.

Almost all suspected third infections occurred after 31 October 2021, i.e., during the period of Omicron circulation. The distribution of times between successive positive tests for individuals’ second and third infections has peaks corresponding to those whose second infections occurred in the second and third waves.

## Individuals with multiple suspected reinfections

**Fig. 3.**
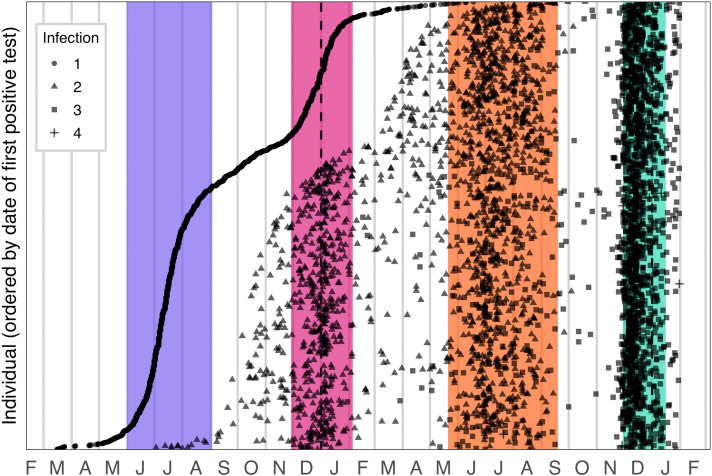
Timing of infections for individuals with multiple suspected reinfections. Circles represent the first positive test of the first detected infection; triangles represent the first positive test of the suspected second infection; squares represent the first positive test of the suspected third infection; crosses represent the first positive test of the suspected fourth infection. Colored bands represent wave periods, defined as the period for which the 7-day moving average of cases was at least 15% of the corresponding wave peak (purple = wave 1, pink = wave 2, orange = wave 3, turquoise = wave 4).

1,778 individuals were identified who had three or more suspected infections. Prior to the emergence of Omicron, most of these individuals initially tested positive during the first wave, with suspected reinfections associated with waves two and three; however, 1,492 individuals with multiple reinfections (83.9%) experienced their third infection after 31 October 2021, which suggests that most third infections are associated with transmission of the Omicron variant (Fig. 3).

## Population-level reinfection trends in South Africa

The population at risk of reinfection has risen monotonically since the beginning of the epidemic, with relatively rapid increases associated with each wave (delayed by 90 days because of our definition of reinfection; Fig. 1B). No suspected reinfections were detected until 23 June 2020, after which the number of suspected reinfections increased gradually. The 7-day moving average of suspected second infections reached a peak of approximately 160 during the second epidemic wave and 350 during the third wave (Fig. 1). Following the third wave, the number of reinfections began to increase dramatically in mid-November 2021. During the fourth wave, the 7-day moving average of suspected second infections reached nearly 2,700 and the 7-day moving average of all suspected reinfections (including second, third, and fourth infections) reached approximately 2,750.

## Comparison of data to projections from a null model

We developed a catalytic model to project the expected number of reinfections through time under the assumption of a constant reinfection hazard coefficient (i.e., a null model of no change in reinfection risk). The model assumes the reinfection hazard is proportional to the 7-day moving average of the total number of diagnosed infections (primary infections and reinfections). During our early monitoring of reinfection risk, we fitted the reinfection hazard coefficient to data from 02 June 2020 to 30 September 2020 to parameterize the null model of no change in the reinfection hazard coefficient through time, and projected the number of reinfections through 30 June 2021. Based on this, we concluded there was no population-level evidence of immune escape and recommended on-going monitoring of reinfection trends (*13*).

**Fig. 4.**
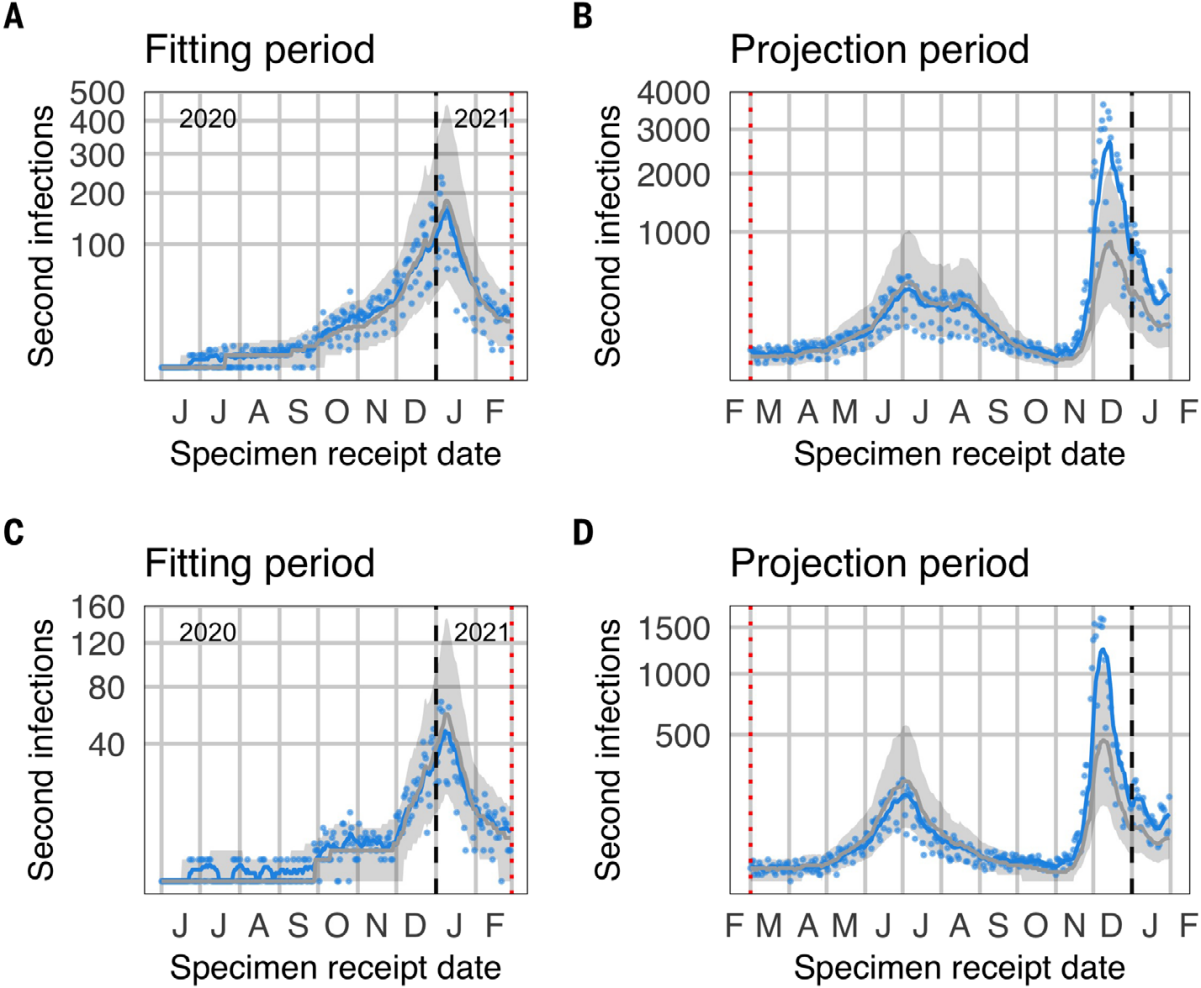
Observed and expected temporal trends in reinfection numbers. Blue lines (points) represent the 7-day moving average (daily values) of suspected reinfections. Grey lines (bands) represent median predictions (95% projection intervals) from the null model. (**A** and **C**) The null model was fit to data on suspected reinfections through 28 February 2021. (**B** and **D**) Comparison of data to projections from the null model over the projection period. The divergence observed reinfections from the projection interval in November suggests immune escape. (A) and (B): National. (C) and (D) Gauteng.

Given that there was no evidence of divergence from the null projection during the second wave, and to improve convergence of the MCMC fitting algorithm, for the present analysis, we repeated the fitting process using a window of 02 June 2020 to 28 February 2021 (representing the end of the month in which the second wave ended). This led to good convergence with regard to estimation of both the negative binomial dispersion parameter and the reinfection hazard coefficient (fig. S4) and allowed us to fit the model to all nine provinces. The 7-day moving average of observed reinfections and most individual daily values fall within the projection interval from the beginning of the projection period though the end of the third wave (Fig. 4). From early November 2021, however, the 7-day moving average of observed reinfections reached the upper bound of the projection interval, with many individual daily numbers falling well above the projection interval, both nationally and in Gauteng (Fig. 4). This observed deviation from the projection under the null model is a signature of immune evasion, and the timing of this deviation suggests it is associated with the emergence of the Omicron variant. A similar pattern has now been seen across all provinces (figs. S5 to S7).

## Estimation of time-varying infection and reinfection hazards

**Fig. 5.**
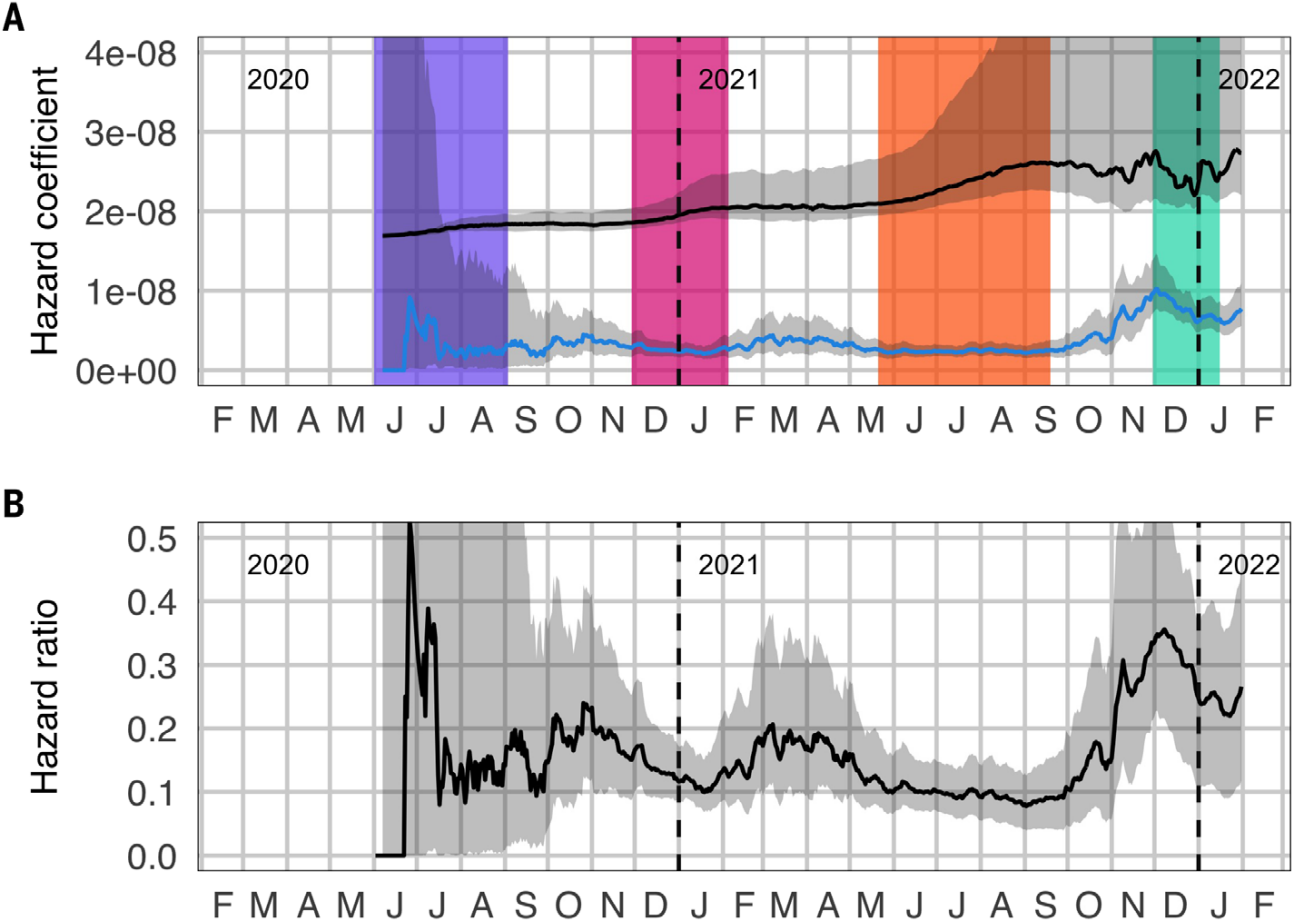
Estimates of infection and reinfection hazards. (**A**) Estimated time-varying hazard coefficients for primary infection (black) and second infections (blue). Colored bands represent wave periods, defined as the period for which the 7-day moving average of cases was at least 15% of the corresponding wave peak (purple = wave 1, pink = wave 2, orange = wave 3, turquoise = wave 4). (**B**) Ratio of the estimated hazard for reinfections to the estimated hazard for primary infections.

We also examined changes in the reinfection risk via a method that relies on reconstruction of the numbers of observed and unobserved first and second infections through time (see Materials and Methods for details). Based on this approach, the estimated hazard coefficient for primary infection increased steadily through the end of the third wave, as expected under a combination of relaxing of restrictions, behavioral fatigue, and introduction of variants with increased transmissibility (Beta and Delta). The estimated hazard coefficient for reinfection, in contrast, remained relatively constant throughout this period, with the exception of an initial spike in mid-2020 (Fig. 5). Because both reinfection numbers and the population eligible for reinfection were very low at the time, this increase may be an artifact of intense follow-up of the earliest cases or simply noise due to small numbers. The estimated mean ratio of reinfection hazard to primary infection hazard decreased slightly from 0.15 in wave 1 to 0.12 in wave 2 and 0.09 in wave 3. The absolute values of the hazard coefficients and hazard ratio are sensitive to assumed observation probabilities for primary infections and reinfections; however, the temporal trends are robust (fig. S8).

The picture changed following the end of the third wave. Although there is substantial uncertainty in the estimated hazard coefficient for primary infection, it appeared to decrease from early October 2021, with a simultaneous increase in the estimated reinfection hazard coefficient (Fig. 5). This change became more marked from the beginning of November, with the mean ratio of reinfection hazard to primary infection hazard for the period from 01 November 2021 to the beginning of the fourth wave increasing to 0.25, and a mean ratio during the fourth wave of 0.27.

These findings are consistent with the estimates from a generalized linear mixed model based on the reconstructed data set. In this analysis, the relative hazard ratio for wave 2 versus wave 1 was 0.71 (CI_95_: 0.60–0.85) and for wave 3 versus wave 1 was 0.54 (CI_95_: 0.45–0.64). The relative hazard ratio for the period of Omicron emergence (01 November 2021 to the start of the fourth wave) versus wave 1 was 1.75 (CI_95_: 1.48–2.10), and for wave 4 versus wave 1 was 1.70 (CI_95_: 1.44–2.04).

## Discussion and limitations

Our analyses suggest that the cumulative number of reinfections observed through the end of wave 3 was consistent with the null model of no change in reinfection risk through time. Furthermore, our findings suggest that the relative hazard of reinfection versus primary infection decreased with each subsequent wave of infections through September 2021, as would be expected if the risk of primary infection increased without a corresponding increase in reinfection risk. Thus, our analyses show no population-level evidence of immune escape associated with emergence of the Beta or Delta variants. In contrast, in November 2021, the number of daily new reinfections spiked and exceeded the 95% projection interval from the null model, accompanied by a notable increase in the hazard ratio for reinfection versus primary infection. The timing of these changes strongly suggests that they were driven by the emergence of the Omicron variant. This finding has now been supported by analyses of reinfection with Omicron in the United Kingdom and Qatar (*14*–*16*).

Differences in the time-varying force of infection, original and subsequent circulating lineages, testing strategies, and vaccine coverage limit the usefulness of direct comparisons of rates of reinfections across countries or studies. Pre-Omicron reinfection does however appear to be relatively uncommon. The PCR-confirmed reinfection rate ranged from 0% – 1.1% across eleven studies included in a systematic review (*17*). While none of the studies included in the systematic review reported increasing risk of reinfection over time, the duration of follow-up was less than a year and most studies were completed prior to the identification of variants of concern; furthermore, all studies predated the emergence of Omicron. Our findings for the period prior to the emergence of Omicron are consistent with results from the PHIRST-C community cohort study conducted in two locations in South Africa, which found that infection prior to the second wave provided 84% protection against reinfection during the second (Beta) wave (*18*), comparable to estimates of the level of protection against reinfection for wild type virus from the SIREN study in the UK (*19*).

A preliminary analysis of reinfection trends in England suggested that the Delta variant may have a higher risk of reinfection compared to the Alpha variant (*20*); however, this analysis did not take into account the temporal trend in the population at risk for reinfection, which may have biased the findings. Because the Alpha variant never dominated transmission in South Africa, we are unable to analyze the relative risk of reinfection for the Alpha and Delta variants in this context; however, data from Qatar suggest that protection provided by prior infection is similar for Alpha and Delta (*14*).

Our findings regarding the Beta and Delta variants are somewhat at odds with in vitro neutralization studies. Both the Beta and Delta variants are associated with decreased neutralization by some anti-receptor binding-domain (anti-RBD) and anti-N-terminal domain (anti-NTD) monoclonal antibodies though both Beta and Delta each remain responsive to at least one anti-RBD (*8*, *9*, *21*). In addition, Beta and Delta are relatively poorly neutralized by convalescent sera obtained from unvaccinated individuals infected with non-VOC virus (*7*–*9*, *21*). Lastly sera obtained from individuals after both one and two doses of the BNT162b2 (Pfizer) or ChAdOx1 (AstraZeneca) vaccines displayed lower neutralization of the Beta and Delta variants when compared to non-VOC and Alpha variant (*9*); although this does not have direct bearing on reinfection risk, it is an important consideration for evaluating immune escape more broadly. Non-neutralizing antibodies and T-cell responses could explain the apparent disjuncture between our findings and the in vitro immune evasion demonstrated by both Beta and Delta.

### Strengths of this study

Our study has three major strengths. First, we analyzed a large routine national data set comprising all confirmed cases in the country, allowing a comprehensive analysis of suspected reinfections in the country. Second, we found consistent results using two different analytical methods, both of which accounted for the changing force of infection and increasing numbers of individuals at risk for reinfection. Third, our real-time routine monitoring was sufficient to detect a population-level signal of immune evasion during the initial period of emergence of the Omicron variant in South Africa, prior to results from laboratory-based neutralization tests, providing timely information of importance to global public health planning.

### Limitations of this study

The primary limitation of this study is that changes in testing practices, health-seeking behavior, or access to care have not been accounted for in these analyses. Estimates based on serological data from blood donors suggest substantial geographic variability in detection rates (*22*), which may contribute to the observed differences in reinfection patterns by province (fig. S1). Detection rates likely also vary through time and by other factors affecting access to testing, which may include occupation, age, and socioeconomic status. In particular, rapid antigen tests, which were introduced in South Africa in late 2020, may be under-reported despite mandatory reporting requirements. Although we have incorporated adjustments that account for late reporting of antigen tests, if under-reporting of antigen tests was substantial and time-varying it could still influence our findings. However, comparing temporal trends in infection risk among those eligible for reinfection with the rest of the population, as in approach 2, mitigates against potential failure to detect a substantial increase in risk.

Civil unrest during July 2021 severely disrupted testing in Gauteng and KwaZulu-Natal, the two most populous provinces in the country. Case data are unreliable during the period of unrest and a key assumption of our models - that the force of infection is proportional to the number of positive tests - was violated during this period, resulting in increased misclassification of individuals regarding their status as to whether they are at risk of primary or re-infection. The effect of this misclassification on the signal of immune escape during the period of Omicron’s emergence would likely be small and would be expected to bias subsequent reinfection hazard estimates downwards.

The purpose of our analysis is to detect changes in the relative reinfection risk through time, rather than to precisely estimate what the reinfection risk is at any particular point in time. While issues related to underdetection of both primary infections and reinfections are likely to affect the projection intervals against which we compare observed reinfections, we believe that our assessment of changes in the reinfection hazard are fairly robust to these detection issues. In effect, Approach 1 follows an open cohort of individuals who have had a first detected infection. Through time, this may include an increasing number of individuals whose first true infection was missed and whose first diagnosed infection is in fact a reinfection. These individuals would presumably be at a reduced risk of acquiring a new infection relative to those whose first detected infection was their first true infection. Two other factors would bias the results in the same direction: undetected reinfections in the cohort of individuals having had a first detected infection and deaths within this cohort, which are not accounted for due to not having a mortality line list that can be linked to the positive test data. All three factors artificially inflate the estimated denominator of individuals at risk for a second detected infection, thereby reducing the apparent reinfection risk. These factors may explain the slightly lower observed than projected number of reinfections throughout the Delta wave but did not have a substantial enough effect to prevent detection of the increased reinfection risk associated with the Omicron variant.

The other main limitation of this study is that reinfections were not confirmed by sequencing or by requiring a negative test between putative infections. Nevertheless, the 90-day window period between consecutive positive tests reduces the possibility that suspected reinfections were predominantly the result of prolonged viral shedding. Furthermore, due to data limitations, we were unable to examine whether symptoms and severity in primary episodes correlate with protection against subsequent reinfection.

Lastly, while vaccination may increase protection in previously infected individuals (*23*–*26*), vaccination coverage in South Africa was very low during much of the study period, with 22.5% of the population fully vaccinated by 30 November 2021 (*27*). Nevertheless, increasing vaccination uptake may reduce the risks of both primary infection and reinfection. The vaccination status of individuals with suspected reinfections identified in this study was unknown. Application of our approach to other locations with higher vaccine coverage would require a more nuanced consideration of the potential effect of vaccination. Further areas for future methodological development include accounting for potential waning of natural and vaccine-derived immunity, as well as methods to track changes in the risk of multiple (three or more) infections.

Given the limitations outlined above, estimates of the extent of immune evasion based on our approach, which aims to detect changing trends rather than make precise estimates, should be treated with caution.

## Conclusion

We find evidence of a substantial increase in the risk of reinfection that is temporally consistent with the timing of the emergence of the Omicron variant in South Africa, suggesting that Omicron’s selection advantage is at least partially driven by an increased ability to infect previously infected individuals.

In contrast, we find no evidence that reinfection risk increased as a result of the emergence of Beta or Delta variants, suggesting that the selective advantage that allowed these variants to spread derived primarily from increased transmissibility, rather than immune evasion. The discrepancy between the population-level evidence presented here and expectations based on laboratory-based neutralization assays for Beta and Delta highlights the need to identify better correlates of immunity for assessing immune escape in vitro.

Immune evasion from prior infection has important implications for public health globally. As new variants emerge, methods to quantify the extent of immune evasion for both natural and vaccine-derived immunity, as well as changes in transmissibility and disease severity, will be urgent priorities to inform facility readiness planning and other public health operations.

## Methods

### Data sources

Data analyzed in this study came from two sources maintained by the National Institute for Communicable Diseases (NICD): the outbreak response component of the Notifiable Medical Conditions Surveillance System (NMC-SS) deduplicated case list and the line list of repeated SARS-CoV-2 tests. All positive tests conducted in South Africa appear in the combined data set, regardless of the reason for testing or type of test (PCR or antigen detection), and include the large number of positive tests that were retrospectively added to the data set on 23 November 2021 (*28*). We note that, of the 18,585 cases reported on 23 November, 93% had a specimen receipt date before 01 November 2021, and 6% had specimen receipt dates on or after 21 November 2021.

A combination of deterministic (national identity number, names, dates of birth) and probabilistic linkage methods were utilized to identify repeated tests conducted on the same person. In addition, provincial COVID-19 contact tracing teams identify and report repeated SARS-CoV-2 positive tests to the NICD, whether detected via PCR or antigen tests. The unique COVID-19 case identifier which links all tests from the same person was used to merge the two datasets. Irreversibly hashed case IDs were generated for each individual in the merged data set.

Primary infections and suspected repeat infections were identified using the merged data set. Repeated case IDs in the line list were identified and used to calculate the time between consecutive positive tests for each individual, using specimen receipt dates. If the time between sequential positive tests was at least 90 days, the more recent positive test was considered to indicate a suspected new infection. We present a descriptive analysis of suspected third and fourth infections, although only suspected second infections were considered in the analyses of temporal trends. Incidence time series for primary infections and reinfections are calculated by specimen receipt date of the first positive test associated with the infection, and total observed incidence is calculated as the sum of first infections and reinfections. The specimen receipt date was chosen as the reference point for analysis because it is complete within the data set; however, problems have been identified with accuracy of specimen receipt dates for tests associated with substantially delayed reporting from some laboratories. For these tests, which had equivalent entries for specimen receipt date and specimen report date that were more than 7 days after the sample collection date, the specimen receipt date was adjusted to be 1 day after the sample collection date, reflecting the median delay across all tests.

All analyses were conducted in the R statistical programming language (R version 4.0.5 (2021-03-31)).

### Timing of reinfections

We calculated the time between successive infections as the number of days between the last positive test associated with an individual’s first identified infection (i.e., within 89 days of a previous positive test, if any) and the first positive test associated with their suspected subsequent infection (i.e., at least 90 days after the most recent positive test). We analyzed the distribution of these times for all second and third infections, and for the subset of second and third infections occurring since 01 November 2021.

### Statistical analysis of reinfection trends

We analyzed the NICD national SARS-CoV-2 routine surveillance data to evaluate whether reinfection risk has changed since emergence of variants of concern in South Africa. We evaluated the daily numbers of suspected reinfections using two approaches. First, we constructed a simple null model based on the assumption that the reinfection hazard experienced by previously diagnosed individuals is proportional to the incidence of detected infections and fit this model to the pattern of suspected second infections observed through 28 February 2021. The null model assumes no change in the reinfection hazard coefficient through time. We then compared observed reinfections after the fitting period to expected reinfections under projections from the null model.

Second, we evaluated whether there has been a change in the relative hazard of reinfection versus primary infection, to distinguish between increased overall transmissibility of the variants and any additional risk of reinfection due to potential immune escape. To do this, we calculated a hazard coefficient at each time point for primary and second infections and compared their relative values through time.

### Approach 1: Catalytic model assuming a constant reinfection hazard coefficient

#### Model description

For a case testing positive on day t (by specimen receipt date), we assumed the reinfection hazard is 0 for each day from t+1 to t+89 and $\lambda {\hat{I}}_{\tau }$ for each day τ≥t+90 , where ${\hat{I}}_{\tau }$ is the 7-day moving average of the detected case incidence (first infections and reinfections) for day τ . The probability of a case testing positive on day t having a diagnosed reinfection by day x is thus $p\left(t,x\right)=1-e^{-\overset{i=x}{\underset{i=t+90}{\sum }}\lambda {\hat{I}}_{i}}$ , and the expected number of cases testing positive on day t that have had a diagnosed reinfection by day x is $I_{t}^{1}p\left(t,x\right)$ , where $I_{t}^{1}$ is the detected case incidence (putative first infections only) for day t . Thus, the expected cumulative number of reinfections by day x is $Y_{x}=\overset{t=x}{\underset{t=0}{\sum }}I_{t}^{1}p\left(t,x\right)$ , and the expected daily incidence of reinfections on day x is $D_{x}=Y_{x}-Y_{x-1}$ .

#### Model fitting

The model was fitted to observed reinfection incidence through 28 February 2021 assuming data are negative binomially distributed with mean $D_{x}$ . The reinfection hazard coefficient ( λ ) and the inverse of the negative binomial dispersion parameter ( κ ) were fitted to the data using a Metropolis-Hastings Monte Carlo Markov Chain (MCMC) estimation procedure implemented in the R Statistical Programming Language. We ran 4 MCMC chains with random starting values for a total of 10,000 iterations per chain, discarding the first 1,000 iterations (burn-in). Convergence was assessed using the Gelman-Rubin diagnostic (*29*).

#### Model-based projection

We used 1,500 samples from the joint posterior distribution of fitted model parameters to simulate possible reinfection time series under the null model, generating 100 stochastic realizations per parameter set. We then calculated projection intervals as the middle 95% of daily reinfection numbers across these simulations.

We applied this approach at the national and provincial levels.

### Approach 2: Estimation of time-varying infection and reinfection hazards

We estimated the time-varying empirical hazard of infection as the daily incidence per susceptible individual. This approach requires reconstruction of the number of susceptible individuals through time. We distinguish between three “susceptible” groups: naive individuals who have not yet been infected ( $S_{1}$ ), previously infected individuals who had undetected infections at least 90 days ago and have not yet had a second infection ( $S_{2}^{u}$ ), and previously infected individuals who had a prior positive test at least 90 days ago and have not yet had a second infection ( $S_{2}$ ). We estimate the numbers of individuals in each of these categories on day t as follows:

$$S_{1}\left(t\right)=N-\overset{i=t}{\underset{i=0}{\sum }}P\left(t\right)$$

where N is the total population size and $P\left(t\right)=P_{obs}\left(t\right)/p_{obs}$ is the total number of primary infections on day t , of which $P_{obs}\left(t\right)$ were observed and $P_{missed}\left(t\right)=P\left(t\right)-P_{obs}\left(t\right)$ were missed.

$$S_{2}^{u}\left(t\right)=\overset{t-89}{\underset{i=0}{\sum }}P_{missed}\left(i\right)-\overset{t-1}{\underset{i=0}{\sum }}U\left(i\right)$$

where $U\left(t\right)=h_{2}\left(t\right)S_{2}^{u}\left(t\right)$ is the number of new infections among individuals whose first infection was missed. These individuals are assumed to experience the same infection hazard as individuals whose primary infection was diagnosed and who have not yet been reinfected, estimated as $h_{2}\left(t\right)=\frac{{\hat{X}}_{t}/p_{obs_{2}}}{S_{2}\left(t\right)}$ . Because individuals are not eligible for reinfection until at least 90 days after their primary infection, we set $U\left(t\right)=h_{2}\left(t\right)=0$ when t<90 .

$$S_{2}\left(t\right)=\overset{i=t-89}{\underset{i=0}{\sum }}P_{obs}\left(i\right)-\overset{i=t}{\underset{i=0}{\sum }}\frac{X_{i}}{p_{obs_{2}}}$$

where $p_{obs_{2}}$ is the probability of detection for individuals who have had a previously identified infection, and $X_{i}$ is the number of individuals with a second detected infection on day i . Only the possibility of second infections are accounted for in the model, which was developed to monitor reinfection risk against a background in which reinfections were rare.

This setup allows recursive calculation of $U\left(t\right)$ and therefore $U_{obs}\left(t\right)=U\left(t\right)p_{obs_{u}}$ , where $p_{obs_{u}}$ is the probability of a second infection being observed in an individual whose first infection was missed, and $P_{obs}\left(t\right)=C_{t}-U_{obs}\left(t\right)$ , where $C_{t}$ is the number of individuals with their first positive test on day t (i.e., detected cases). The daily hazard of infection for previously uninfected individuals is then estimated as $h_{1}\left(t\right)=\frac{{\hat{P}}_{t}}{S_{1}\left(t\right)}$ .

If we assume that the hazard of infection is proportional to the 7-day moving average of infection incidence ( $\hat{Y_{t}}=\hat{P}\left(t\right)+\hat{U}\left(t\right)+\hat{X_{t}}/p_{obs_{2}}$ ), we can then examine the infectiousness of the virus through time as $\lambda_{1}\left(t\right)=h_{1}\left(t\right)/\hat{Y_{t}}$ and $\lambda_{2}\left(t\right)=h_{2}\left(t\right)/\hat{Y_{t}}$ . We constructed uncertainty intervals around $\lambda_{1}\left(t\right)$ , $\lambda_{2}\left(t\right)$ , and their ratio, taking into account both measurement noise and uncertainty in the observation parameters (see the supplementary materials for details).

We also used this approach to construct a data set with the daily numbers of individuals eligible to have a primary infection ( $S_{1}\left(t\right)$ ) or suspected second infection ( $S_{2}\left(t\right)$ ) by wave. Wave periods were defined as the time surrounding the wave peak for which the 7-day moving average of case numbers was above 15% of the wave peak. We then analyzed these data using a generalized linear mixed model to estimate the relative hazard of infection in the population eligible for suspected second infection, compared to the hazard in the population not eligible for suspected second infection. For this analysis, we assume $p_{obs}=0.1$ and $p_{obs_{2}}=0.5$ , which falls within the plausible range of observation probabilities (fig. S8).

Our primary regression model was a Poisson model with a log link function, $\text{groupinc}=\text{Poisson}\left(\mu \right)$ :

$$log\left(\mu \right)∼\text{group*wave}+\text{offset}\left(log\left(\text{groupsize}\right)\right)+\left(\text{day}\right)$$

The outcome variable ( groupinc ) was the reconstructed daily number of observed infections in the two groups ( $P_{obs}\left(t\right)$ and $X_{t}$ ). Our main interest for this analysis was in whether the relative hazard was higher in the second wave, third wave, pre-wave period in which Omicron emerged, and/or fourth wave, relative to during the first wave, thus potentially indicating immune evasion. This effect is measured by the interaction term between group and wave. The offset term is used to ensure that the estimated coefficients can be appropriately interpreted as *per capita* rates. We used day as a proxy for force of infection and reporting patterns and examined models where day was represented as a random effect (to reflect that observed days can be thought of as samples from a theoretical population) and as a fixed effect (to better match the Poisson assumptions). As focal estimates from the two models were indistinguishable, we present only the results based on the random effect assumption.

## Supplementary Materials

### Other Supplementary Material for this manuscript includes the following:

MDAR Reproducibility Checklist

## Notes

References (*32*, *33*)

MDAR Reproducibility Checklist
