# Supplementary material for: Increased risk of SARS-CoV-2 reinfection associated with emergence of Omicron in South Africa
Source: Science. 2022 Mar 15:eabn4947. doi: 10.1126/science.abn4947 (PMC8995029; doi:10.1126/science.abn4947)
Supplement: Supplementary file 1 — Supplementary Text Figs. S1 to S9 Tables S1 and S2 [file science.abn4947_sm.pdf]

Science AAAS

Supplementary Materials for

Increased risk of SARS-CoV-2 reinfection associated with emergence of Omicron in South Africa

Juliet R. C. Pulliam *et al*.

Corresponding author: Juliet R. C. Pulliam, pulliam@sun.ac.za

Science **376**, eabn4947 (2022)
DOI: 10.1126/science.abn4947

The PDF file includes:

Supplementary Text
Figs. S1 to S9
Tables S1 and S2
References

Other Supplementary Material for this manuscript includes the following:

MDAR Reproducibility Checklist

Supplementary Text

1. Supplementary Methods

1.1 Data validation and known data issues

To assess validity of the data linkage procedure and thus verify whether individuals identified as having suspected reinfections did in fact have positive test results at least 90 days apart, we conducted a manual review of a random sample of suspected second infections occurring on or before 20 January 2021 (n=585 of 6026; 9.7%). This review compared fields not used for linkages (address, cell-phone numbers, email addresses, facility, and health-care providers) between records in the NMC-SS and positive test line lists. Where uncertainty remained and contact details were available, patients or next-of-kin were contacted telephonically to verify whether the individual had received multiple positive test results.

Of the 585 randomly selected individuals with possible reinfections in the validation sample, 562 (96%) were verified as the same individual based on fields not used to create the linkages; the remaining 23 (4%) were either judged not a match or to have insufficient evidence (details captured by the clinician or testing laboratory) to determine whether the records belonged to the same individual.

Between 5 and 11 December 2022, server issues at the National Health Laboratory Service's Central Data Warehouse prevented one of the identifiers used in the probabilistic linkages from being pulled through into the dataset. Retrospective evaluation of the use of the identifier revealed that it had been required for 0.3% of the links made prior to this time. The impact of this data discrepancy is therefore thought to be minimal.

1.2 Descriptive analysis

We compared the age, gender, and province of individuals with suspected reinfections to individuals eligible for reinfection (i.e., who had a positive test result at least 90 days prior to 31 January 2022).

We did not calculate overall incidence rates by wave because the force of infection is highly variable in space and time, and the period incidence rate is also influenced by the temporal pattern of when people become eligible for reinfection. Incidence rate estimates would therefore be strongly dependent on the time frame of the analysis and not comparable to studies from other locations or time periods.

1.3 Construction of uncertainty intervals for hazard coefficients and hazard ratio

The uncertainty intervals shown in Figure 5 were constructed to take into account both measurement error and uncertainty in the assumed observation probabilities. To capture uncertainty in the observation probabilities, we uniformly sampled 1,000 values from the polygon of plausible values for p_{obs} and p_{obs_2} (Figure S8). For each parameter combination, we used the model described in the main text (approach 2) to reconstruct the numbers of primary and second infections by day, as well as the relevant susceptible populations at risk. We then used each reconstructed data set to construct a 95% confidence interval for the associated Poisson rate, after Sahai and Kurshid (*32*), and for the associated incidence rate ratio, after Ulm (*33*). The confidence limits for the hazard coefficients were approximated by dividing the confidence limits for the Poisson rates by the reconstructed value of the total incidence for each reconstructed data set.

The final uncertainty intervals were then constructed from the distribution of confidence limits based on the 1,000 reconstructed data sets. The median value presented in Figure 5 is the median estimate from across the data sets, and the confidence limits represent the 2.5% and 97.5% quantiles of the lower and upper confidence limits, respectively.

2. Supplementary Results

2.1 Distribution of suspected reinfections by province

Suspected reinfections were identified in all nine provinces (Figure S1). The reinfection rate was highest in Western Cape, where 20,952 of 516,857 eligible primary infections (4.05%) had suspected reinfections and lowest in Northern Cape (2,464 of 92,718; 2.66%). For comparison, the national reinfection rate was 92,718; 3.58% (105,323 of 2,942,248 eligible primary infections). Numbers for all provinces are provided in Table S1.

2.2 Breakdown of suspected reinfections by sex and age group

Among 2,878,217 eligible primary infections with both age and sex recorded, 62,690 of 1,630,428 females (3.85%) and 42,099 of 1,247,789 males (3.37%) had suspected reinfections. Relative to individuals with no identified reinfection, reinfections were concentrated in adults between the ages of 20 and 55 years (Figure S2A). The age distribution of second infections was consistent across waves (Figure S2B). Numbers for all age group-sex combinations are provided in Table S2.

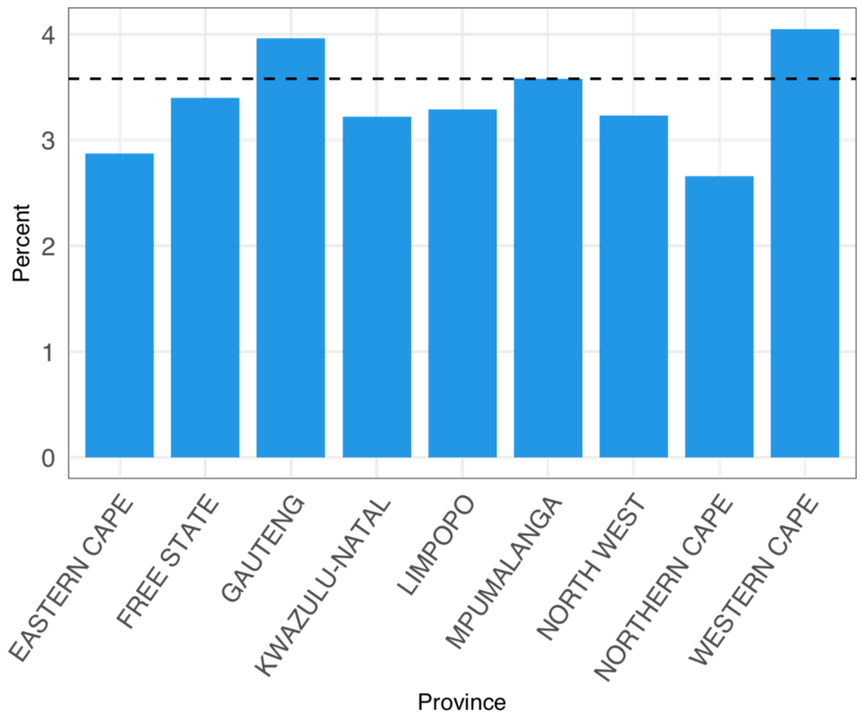

Fig. S1.

Descriptive analysis of suspected reinfections: Percentage of eligible primary infections with suspected reinfections, by province.

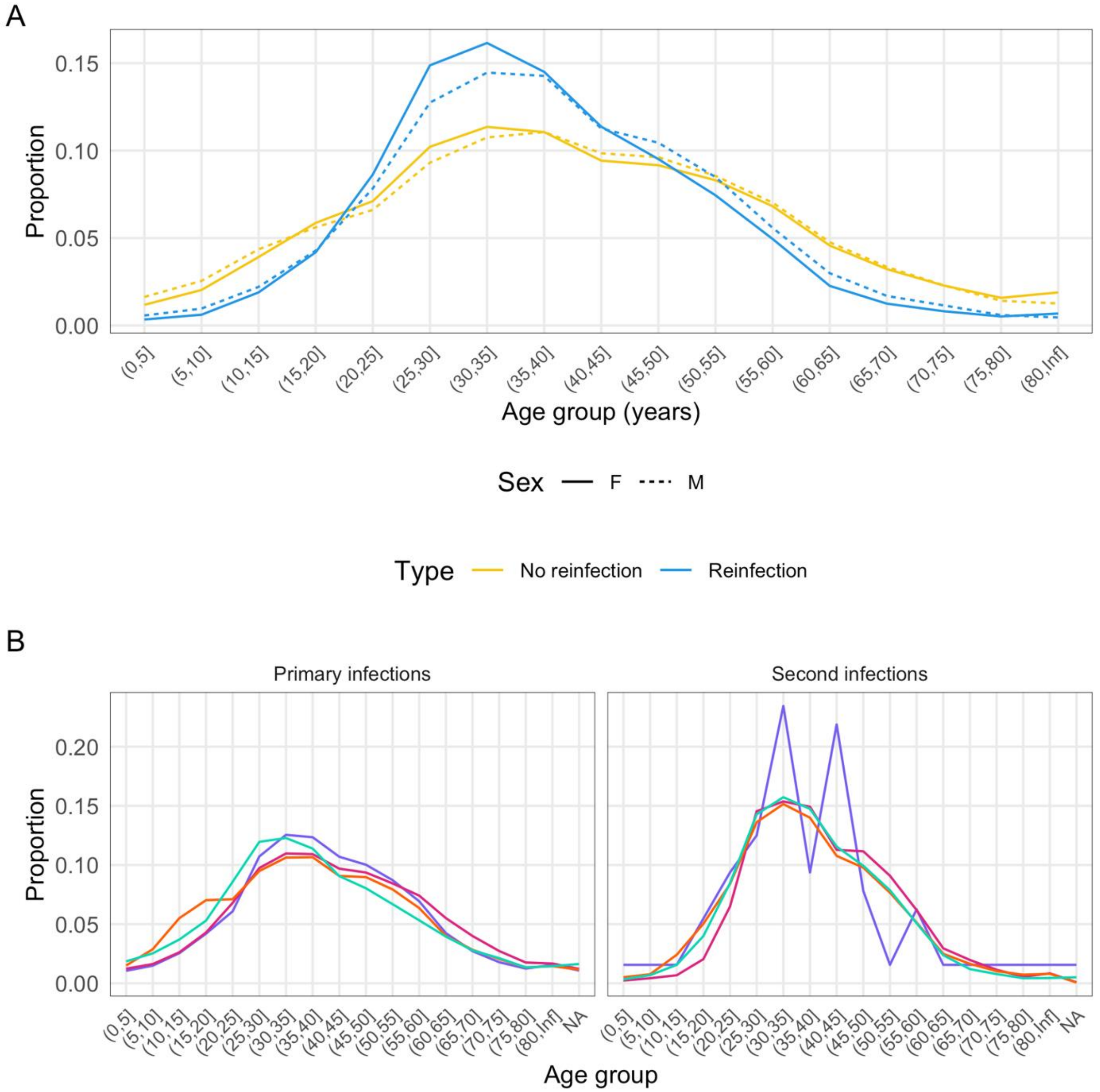

Fig. S2.

Descriptive analysis of suspected reinfections: A: Age distribution of individuals with suspected reinfections (blue) versus eligible individuals with no detected reinfection (yellow), by sex. Solid lines indicate females; dashed lines indicate males. B: Age distribution of primary infections (left) and second infections (right) by wave (purple = wave 1, pink = wave 2, orange = wave 3, turquoise = wave 4).

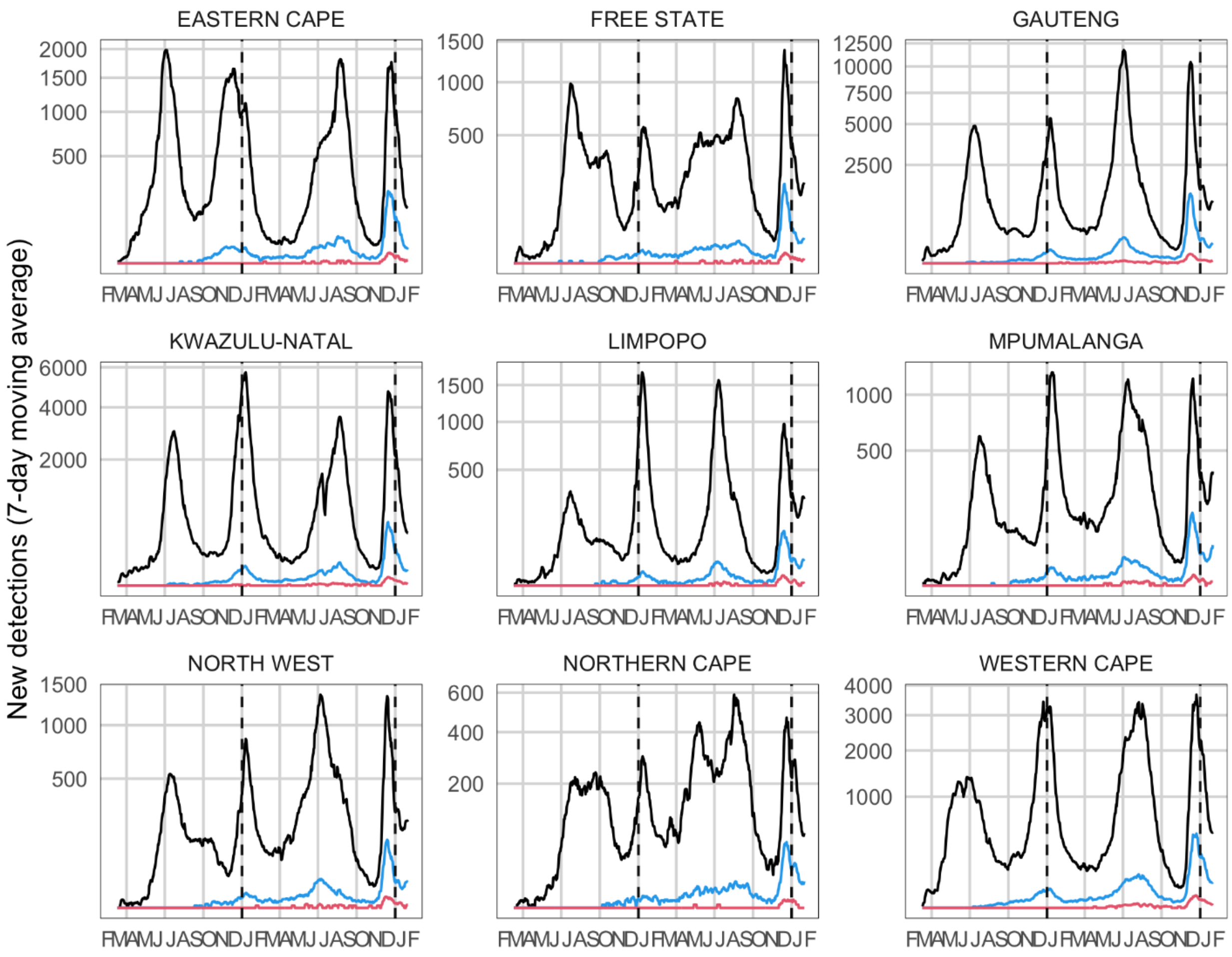

Fig. S3.

Number of detected primary infections (black), suspected reinfections (blue), and suspected third infections (red), by province. Lines represent 7-day moving averages. The y-axes are shown on a square root scale.

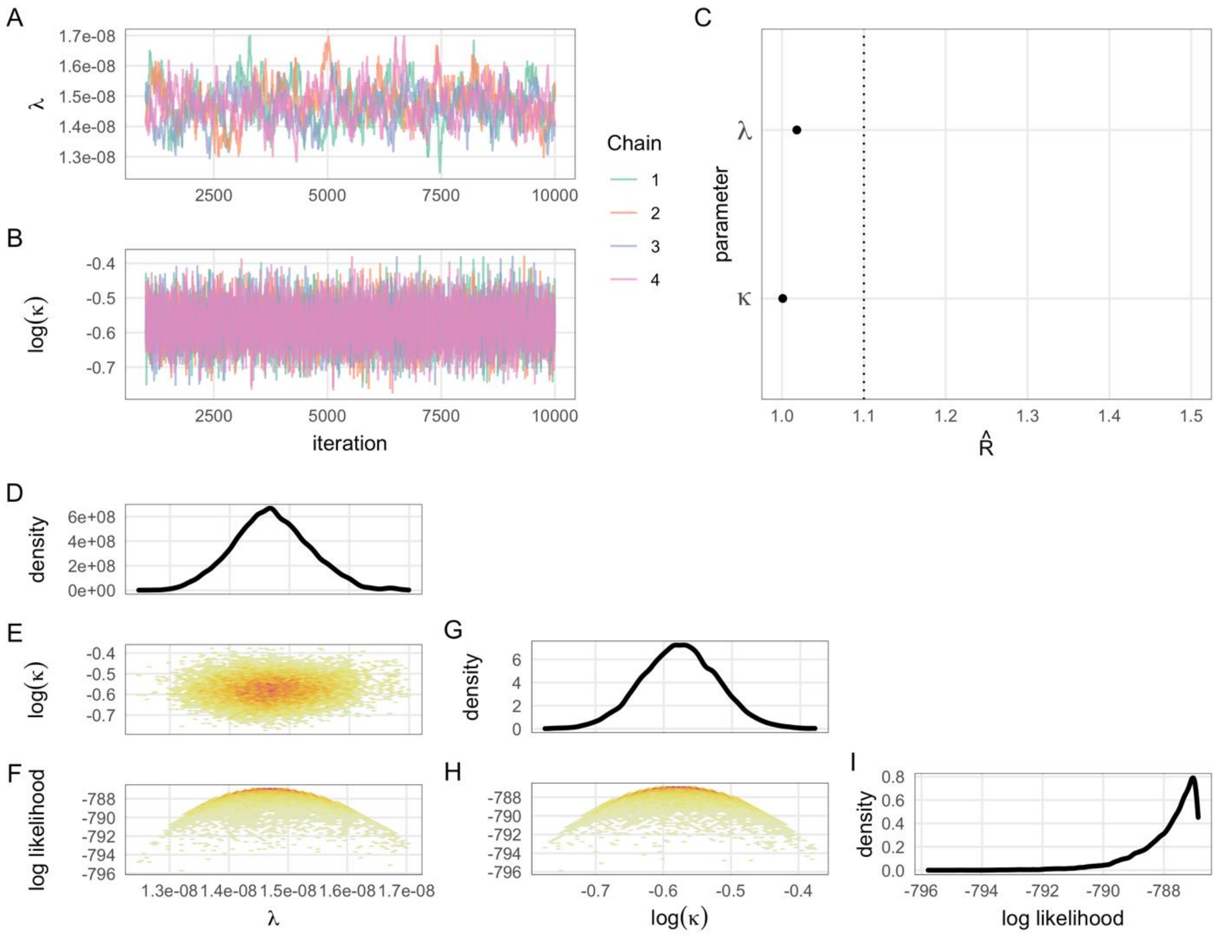

Fig. S4.

Convergence diagnostics and density of the posterior distribution for MCMC fits (approach 1). A and B: MCMC chains for each parameter. C: Gelman-Rubin values (a.k.a. potential scale reduction factors) for each parameter; values less than 1.1 indicate sufficient mixing of chains to suggest convergence. D, G, I: posterior density for each parameter and the log likelihood. E, F, H: 2-D density plots showing correlations between parameters and the log likelihood.

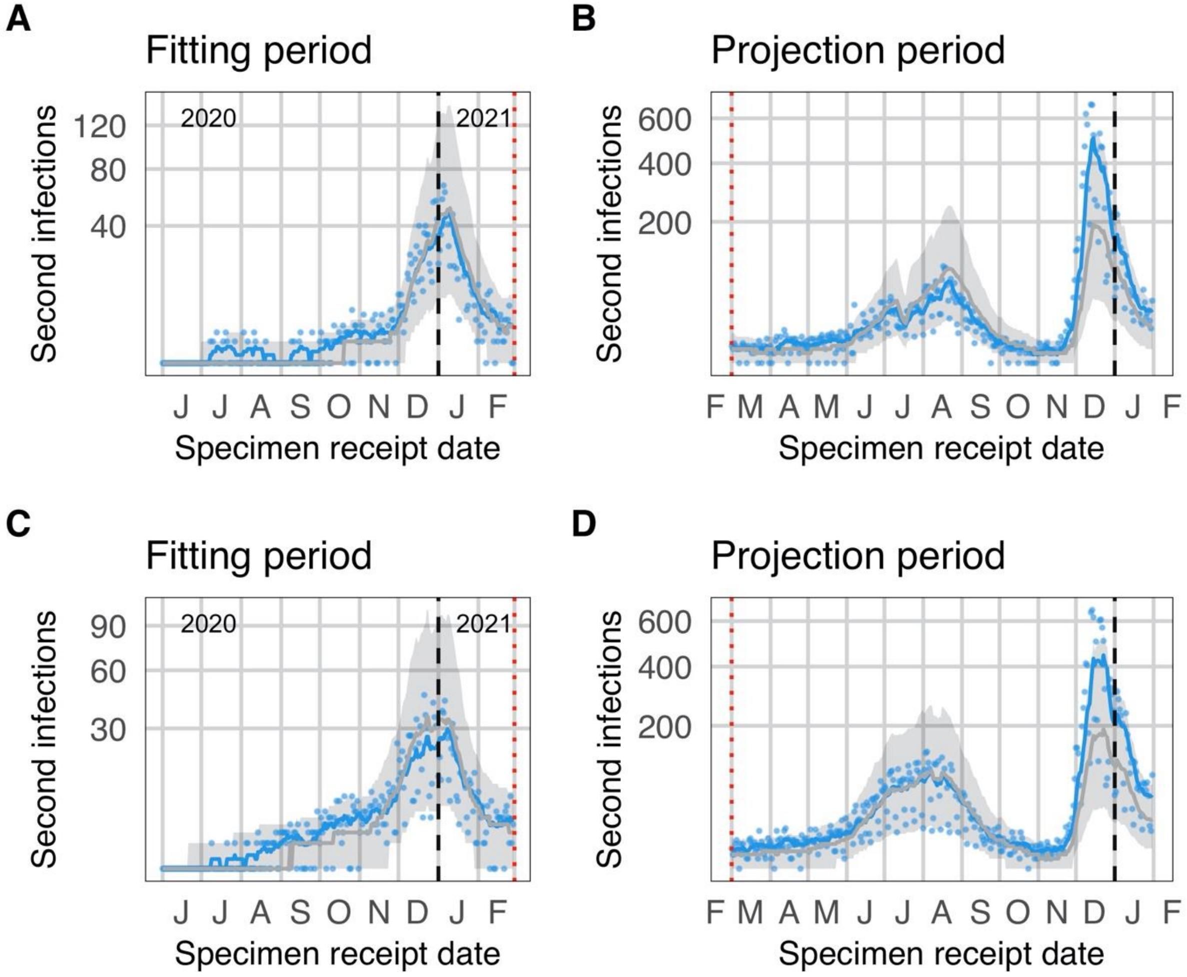

Fig. S5.

Observed and expected temporal trends in reinfection numbers, for the second and third most populous provinces. Blue lines (points) represent the 7-day moving average (daily values) of suspected reinfections. Grey lines (bands) represent mean predictions (95% projection intervals) from the null model. A and B: KwaZulu-Natal. C and D: Western Cape.

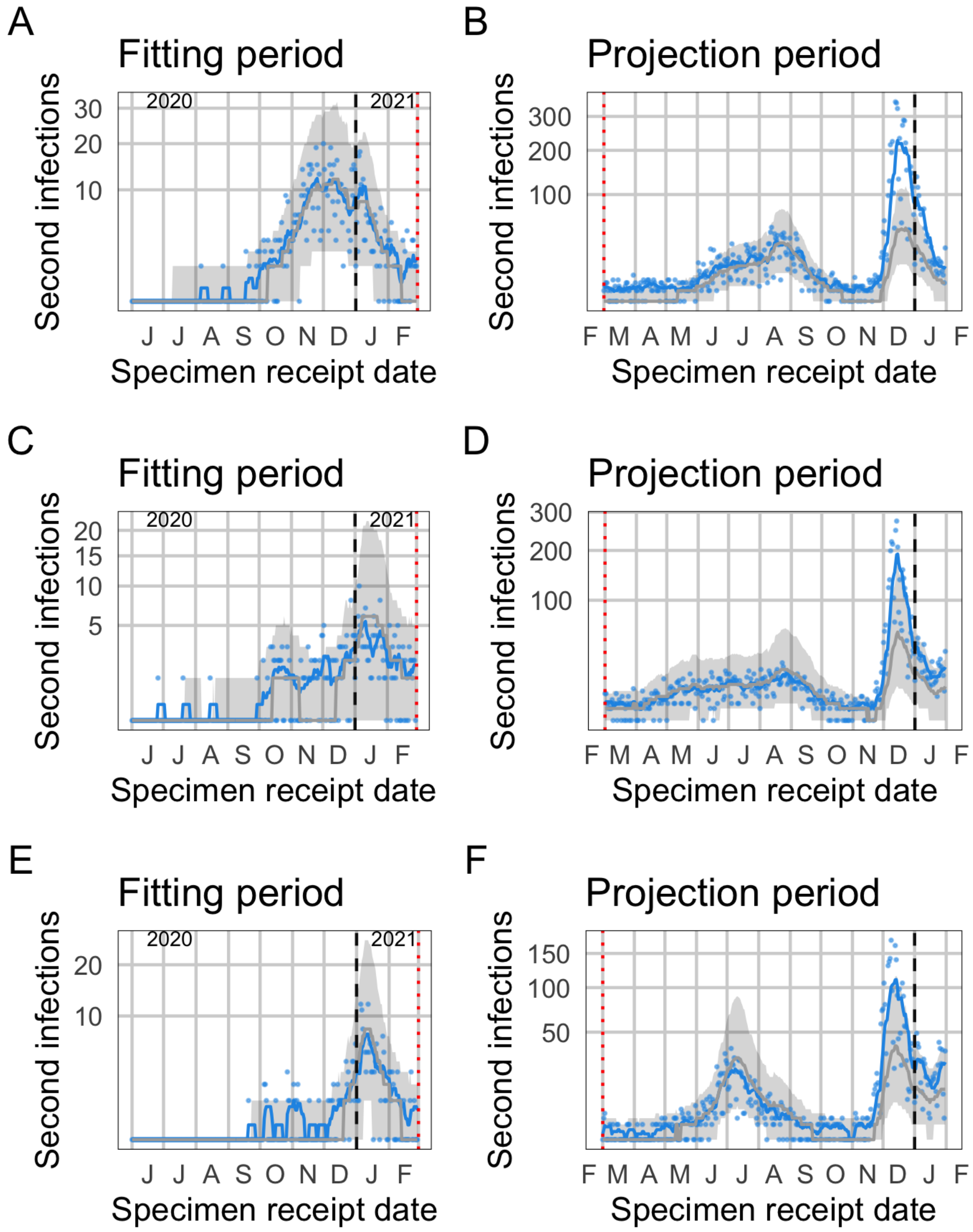

Fig. S6.

Observed and expected temporal trends in reinfection numbers. Blue lines (points) represent the 7-day moving average (daily values) of suspected reinfections. Grey lines (bands) represent mean predictions (95% projection intervals) from the null model. A and B: Eastern Cape. C and D: Free State, E and F: Limpopo.

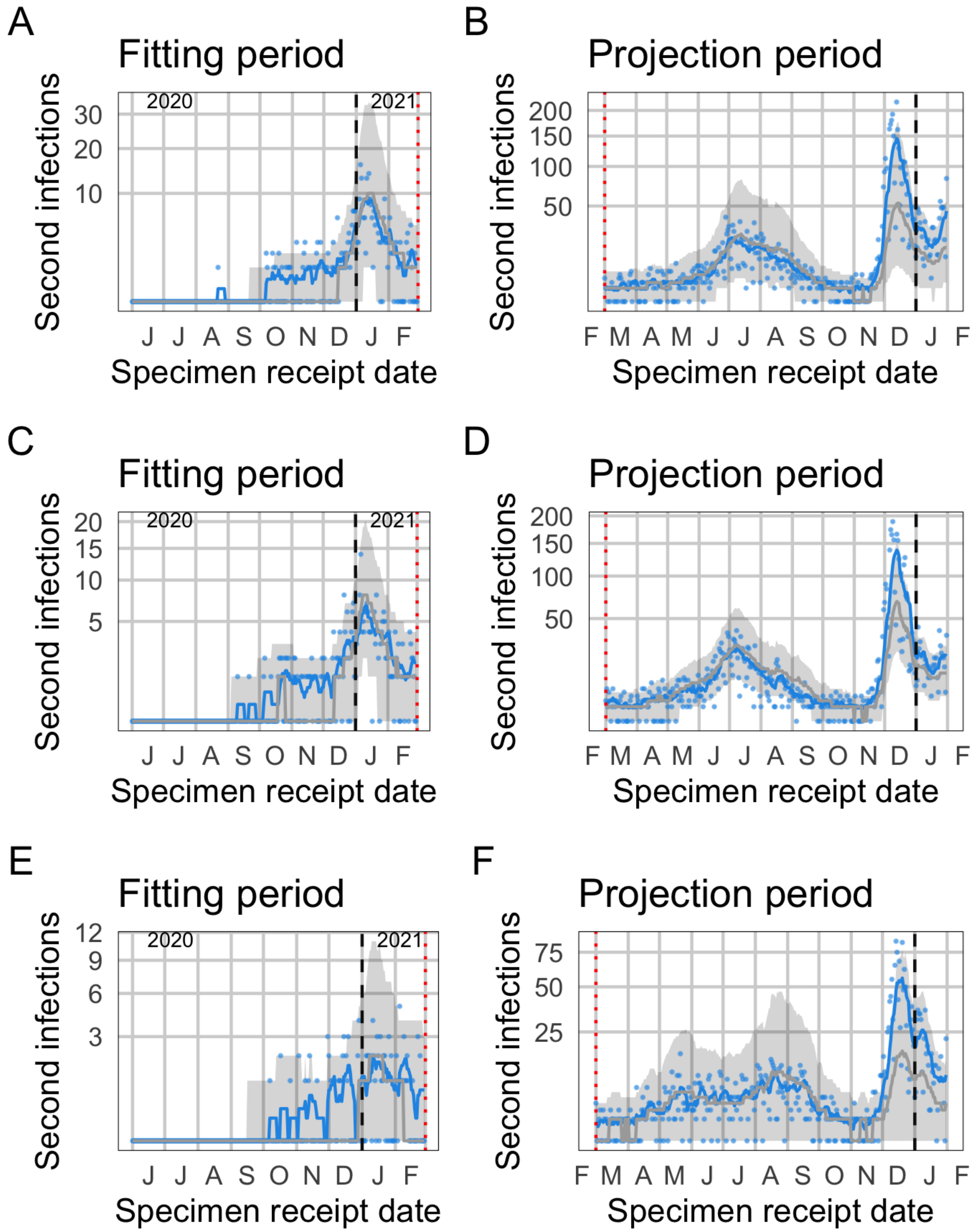

Fig. S7.

Observed and expected temporal trends in reinfection numbers. Blue lines (points) represent the 7-day moving average (daily values) of suspected reinfections. Grey lines (bands) represent mean predictions (95% projection intervals) from the null model. A and B: Mpumalanga. C and D: North West, E and F: Northern Cape.

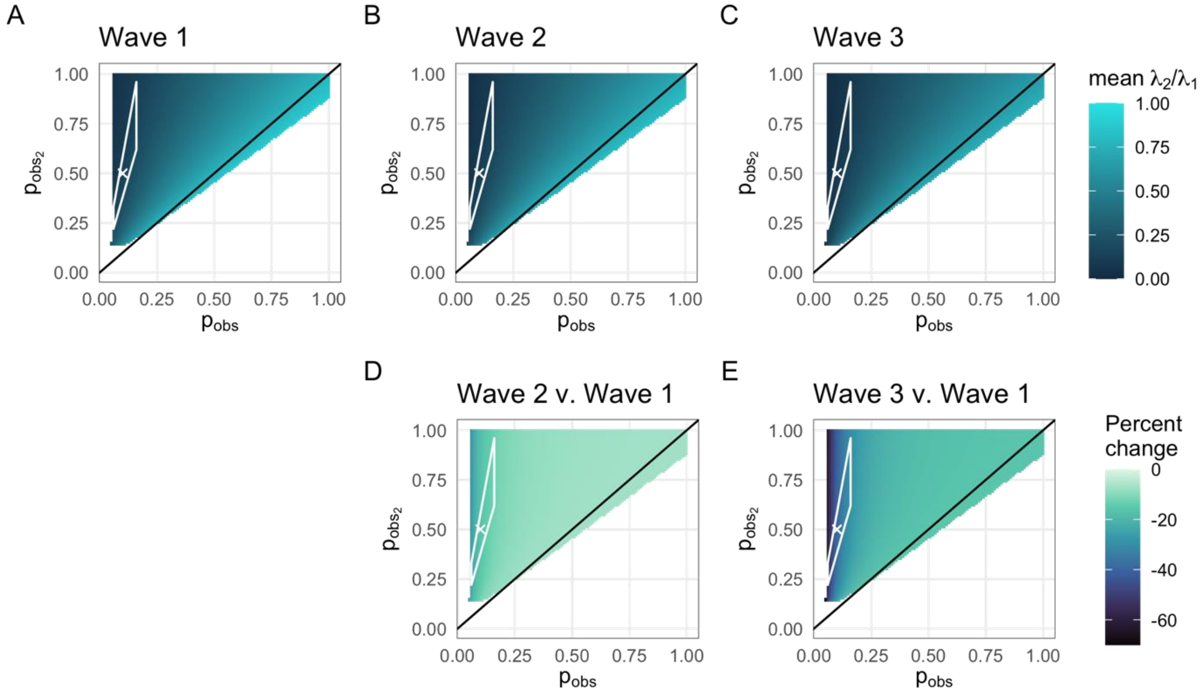

Fig. S8.

Sensitivity analysis of hazard ratio estimates to assumed observation probabilities for primary and second infections. Estimates are shown for the full range of probabilities for which the overall mean relative hazard is between 0 and 1. The white polygon encloses the most plausible estimates (i.e. consistent with relative reinfection risk observed in the SIREN study (*3*) and observation probabilities for primary infection consistent with estimates based on seroprevalence data (*4*)). For all parameter combinations in the plausible range, $p_{obs_2} > p_{obs}$, suggesting that having had a previous test is a good marker for who will test again. Top: Mean relative empirical hazard for reinfections versus primary infections in each wave, as a function of assumed observation probabilities for primary infections (p_{obs}) and reinfections (p_{obs_2}). A: wave 1, B: wave 2, C: wave 3. Bottom: Percent change in the mean relative empirical hazard for reinfections versus primary infections in waves 2 (D) and 3 (E) relative to wave 1, as a function of assumed observation probabilities for primary infections (p_{obs}) and reinfections (p_{obs_2}).

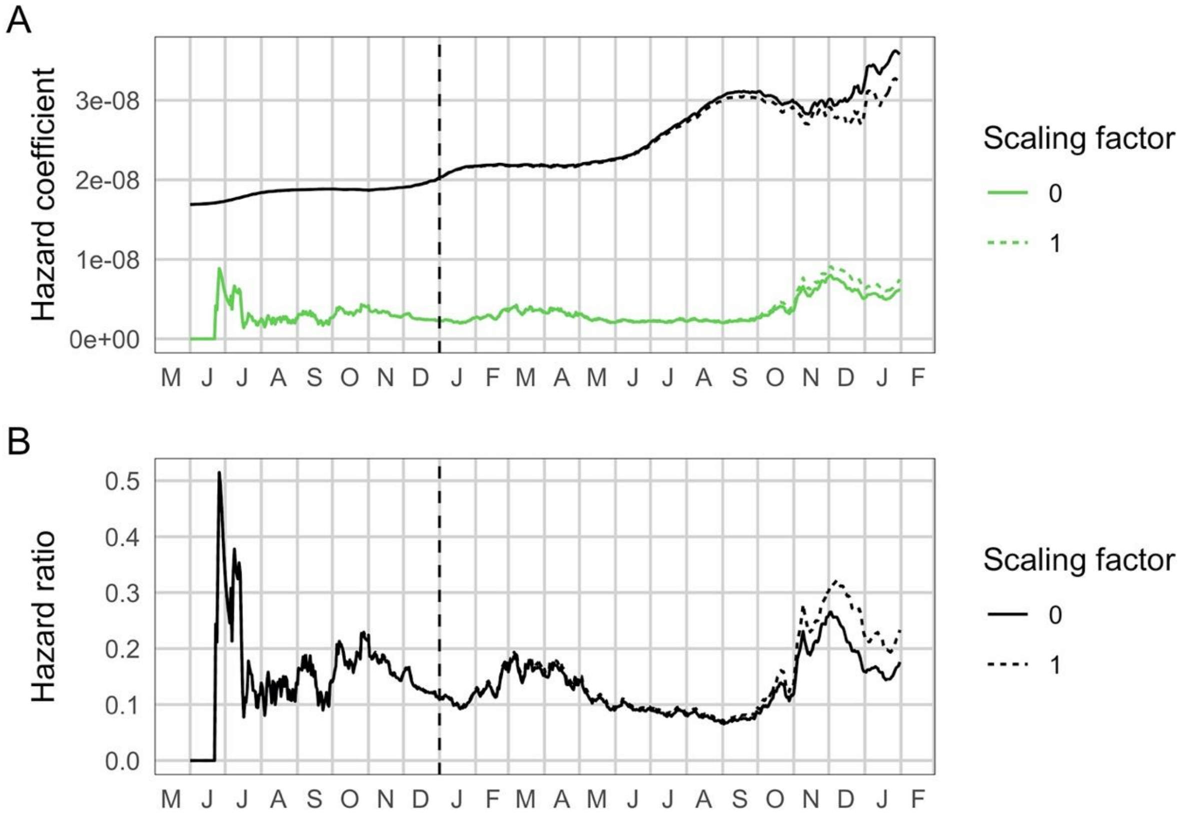

Fig. S9.

Sensitivity analysis of empirical hazard ratio estimates to assumed observation probability for second infections among individuals whose first infection was undetected (p_{obs_u}). In the main analysis we assume that this observation probability is equivalent to other individuals who have not yet had a detected infection ($p_{obs_u} = p_{obs}$, or a scaling factor of 1). Here, we compare this to the case when this probability is equal to 0 ($p_{obs_u} = 0$, or a scaling factor of 0). We consider these to be bounding cases. The figure shown here is for $p_{obs} = 0.1$ and $p_{obs_2} = 0.5$.

Table S1.

Distribution of suspected reinfections by province, South Africa, March 2020 to January 2022.

Province	No reinfection	One reinfection	Two reinfections	Total
EASTERN CAPE	284,397	8,285	127	292,809
FREE STATE	159,055	5,505	87	164,647
GAUTENG	891,193	36,149	597	927,939
KWAZULU-NATAL	501,040	16,409	256	517,705
LIMPOPO	119,323	3,968	87	123,378
MPUMALANGA	147,573	5,394	92	153,059
NORTH WEST	148,183	4,845	106	153,134
NORTHERN CAPE	90,254	2,438	26	92,718
WESTERN CAPE	495,905	20,552	400	516,857
UNKNOWN	2	0	0	2
**Total**	**2,836,925**	**103,545**	**1,778**	**2,942,248**

Table S2.

Breakdown of suspected reinfections by sex and age group (years), South Africa, March 2020 to January 2022.

Sex	Age group	No reinfection	One reinfection	Two reinfections	Total
F	(0,20]	203,687	4,361	50	208,098
F	(20,40]	623,098	33,299	656	657,053
F	(40,60]	528,525	20,505	353	549,383
F	(60,80]	182,785	3,004	33	185,822
F	(80,Inf]	29,643	426	3	30,072
M	(0,20]	170,647	3,353	27	174,027
M	(20,40]	454,922	20,364	397	475,683
M	(40,60]	422,514	14,841	220	437,575
M	(60,80]	142,421	2,673	31	145,125
M	(80,Inf]	15,186	188	5	15,379
	**Total**	**2,773,428**	**103,014**	**1,775**	**2,878,217**

References and Notes

1. H. Tegally, E. Wilkinson, M. Giovanetti, A. Iranzadeh, V. Fonseca, J. Giandhari, D. Doolabh, S. Pillay, E. J. San, N. Msomi, K. Mlisana, A. von Gottberg, S. Walaza, M. Allam, A. Ismail, T. Mohale, A. J. Glass, S. Engelbrecht, G. Van Zyl, W. Preiser, F. Petruccione, A. Sigal, D. Hardie, G. Marais, N. Y. Hsiao, S. Korsman, M.-A. Davies, L. Tyers, I. Mudau, D. York, C. Maslo, D. Goedhals, S. Abrahams, O. Laguda-Akingba, A. Alisoltani-Dehkordi, A. Godzik, C. K. Wibmer, B. T. Sewell, J. Lourenço, L. C. J. Alcantara, S. L. Kosakovsky Pond, S. Weaver, D. Martin, R. J. Lessells, J. N. Bhiman, C. Williamson, T. de Oliveira, Detection of a SARS-CoV-2 variant of concern in South Africa. *Nature* **592**, 438–443 (2021). doi:10.1038/s41586-021-03402-9 Medline

2. Y. Shu, J. McCauley, GISAID: Global initiative on sharing all influenza data - from vision to reality. *Euro Surveill.* **22**, 30494 (2017). doi:10.2807/1560-7917.ES.2017.22.13.30494 Medline

3. R. Viana, S. Moyo, D. G. Amoako, H. Tegally, C. Scheepers, C. L. Althaus, U. J. Anyaneji, P. A. Bester, M. F. Boni, M. Chand, W. T. Choga, R. Colquhoun, M. Davids, K. Deforche, D. Doolabh, L. du Plessis, S. Engelbrecht, J. Everatt, J. Giandhari, M. Giovanetti, D. Hardie, V. Hill, N.-Y. Hsiao, A. Iranzadeh, A. Ismail, C. Joseph, R. Joseph, L. Koopile, S. L. Kosakovsky Pond, M. U. G. Kraemer, L. Kuate-Lere, O. Laguda-Akingba, O. Lesetedi-Mafoko, R. J. Lessells, S. Lockman, A. G. Lucaci, A. Maharaj, B. Mahlangu, T. Maponga, K. Mahlakwane, Z. Makatini, G. Marais, D. Maruapula, K. Masupu, M. Matshaba, S. Mayaphi, N. Mbhele, M. B. Mbulawa, A. Mendes, K. Mlisana, A. Mnguni, T. Mohale, M. Moir, K. Moruisi, M. Mosepele, G. Motsatsi, M. S. Motswaledi, T. Mphoyakgosi, N. Msomi, P. N. Mwangi, Y. Naidoo, N. Ntuli, M. Nyaga, L. Olubayo, S. Pillay, B. Radibe, Y. Ramphal, U. Ramphal, J. E. San, L. Scott, R. Shapiro, L. Singh, P. Smith-Lawrence, W. Stevens, A. Strydom, K. Subramoney, N. Tebeila, D. Tshiabuila, J. Tsui, S. van Wyk, S. Weaver, C. K. Wibmer, E. Wilkinson, N. Wolter, A. E. Zarebski, B. Zuze, D. Goedhals, W. Preiser, F. Treurnicht, M. Venter, C. Williamson, O. G. Pybus, J. Bhiman, A. Glass, D. P. Martin, A. Rambaut, S. Gaseitsiwe, A. von Gottberg, T. de Oliveira, Rapid epidemic expansion of the SARS-CoV-2 Omicron variant in southern Africa. *Nature* 10.1038/s41586-022-04411-y (2022). doi:10.1038/s41586-022-04411-y Medline

4. National Institute for Communicable Diseases, “The daily COVID-19 effective reproductive number (R) in South Africa: Week 47 2021” (National Institute for Communicable Diseases, 2021); https://www.nicd.ac.za/wp-content/uploads/2021/11/COVID-19-Effective-Reproductive-Number-in-South-Africa-week-47.pdf.

5. National Institute for Communicable Diseases, “The daily COVID-19 effective reproductive number (R) in South Africa: Week 51 2021” (National Institute for Communicable Diseases, 2021); https://www.nicd.ac.za/wp-content/uploads/2021/12/COVID-19-Effective-Reproductive-Number-in-South-Africa-week-51.pdf.

6. C. A. B. Pearson, S. P. Silal, M. W. Z. Li, J. Dushoff, B. M. Bolker, S. Abbott, C. van Schalkwyk, N. G. Davies, R. C. Barnard, W. J. Edmunds, J. Bingham, G. Meyer-Rath, L. Jamieson, A. Glass, N. Wolter, N. Govender, W. S. Stevens, L. Scott, K. Mlisana, H. Moultrie, J. R. C. Pulliam, Bounding the levels of transmissibility &

immune evasion of the Omicron variant in South Africa. medRxiv 2021.12.19.21268038 [Preprint] (2021); https://doi.org/10.1101/2021.12.19.21268038.

7. S. Cele, I. Gazy, L. Jackson, S.-H. Hwa, H. Tegally, G. Lustig, J. Giandhari, S. Pillay, E. Wilkinson, Y. Naidoo, F. Karim, Y. Ganga, K. Khan, M. Bernstein, A. B. Balazs, B. I. Gosnell, W. Hanekom, M. S. Moosa, R. J. Lessells, T. de Oliveira, A. Sigal; Network for Genomic Surveillance in South Africa; COMMIT-KZN Team, Escape of SARS-CoV-2 501Y.V2 from neutralization by convalescent plasma. *Nature* **593**, 142–146 (2021). doi:10.1038/s41586-021-03471-w Medline

8. C. K. Wibmer, F. Ayres, T. Hermanus, M. Madzivhandila, P. Kgagudi, B. Oosthuysen, B. E. Lambson, T. de Oliveira, M. Vermeulen, K. van der Berg, T. Rossouw, M. Boswell, V. Ueckermann, S. Meiring, A. von Gottberg, C. Cohen, L. Morris, J. N. Bhiman, P. L. Moore, SARS-CoV-2 501Y.V2 escapes neutralization by South African COVID-19 donor plasma. *Nat. Med.* **27**, 622–625 (2021). doi:10.1038/s41591-021-01285-x Medline

9. D. Planas, D. Veyer, A. Baidaliuk, I. Staropoli, F. Guivel-Benhassine, M. M. Rajah, C. Planchais, F. Porrot, N. Robillard, J. Puech, M. Prot, F. Gallais, P. Gantner, A. Velay, J. Le Guen, N. Kassis-Chikhani, D. Edriss, L. Belec, A. Seve, L. Courtellemont, H. Péré, L. Hocqueloux, S. Fafi-Kremer, T. Prazuck, H. Mouquet, T. Bruel, E. Simon-Lorière, F. A. Rey, O. Schwartz, Reduced sensitivity of SARS-CoV-2 variant Delta to antibody neutralization. *Nature* **596**, 276–280 (2021). doi:10.1038/s41586-021-03777-9 Medline

10. C. Liu, H. M. Ginn, W. Dejnirattisai, P. Supasa, B. Wang, A. Tuekprakhon, R. Nutalai, D. Zhou, A. J. Mentzer, Y. Zhao, H. M. E. Duyvesteyn, C. López-Camacho, J. Slon-Campos, T. S. Walter, D. Skelly, S. A. Johnson, T. G. Ritter, C. Mason, S. A. Costa Clemens, F. Gomes Naveca, V. Nascimento, F. Nascimento, C. Fernandes da Costa, P. C. Resende, A. Pauvolid-Correa, M. M. Siqueira, C. Dold, N. Temperton, T. Dong, A. J. Pollard, J. C. Knight, D. Crook, T. Lambe, E. Clutterbuck, S. Bibi, A. Flaxman, M. Bittaye, S. Belij-Rammerstorfer, S. C. Gilbert, T. Malik, M. W. Carroll, P. Klenerman, E. Barnes, S. J. Dunachie, V. Baillie, N. Serafin, Z. Ditse, K. Da Silva, N. G. Paterson, M. A. Williams, D. R. Hall, S. Madhi, M. C. Nunes, P. Goulder, E. E. Fry, J. Mongkolsapaya, J. Ren, D. I. Stuart, G. R. Screaton, Reduced neutralization of SARS-CoV-2 B.1.617 by vaccine and convalescent serum. *Cell* **184**, 4220–4236.e13 (2021). doi:10.1016/j.cell.2021.06.020 Medline

11. A. Rössler, L. Riepler, D. Bante, D. von Laer, J. Kimpel, SARS-CoV-2 Omicron variant neutralization in serum from vaccinated and convalescent persons. *N. Engl. J. Med.* **386**, 698–700 (2022). doi:10.1056/NEJMc2119236 Medline

12. D. Planas, N. Saunders, P. Maes, F. Guivel-Benhassine, C. Planchais, J. Buchrieser, W.-H. Bolland, F. Porrot, I. Staropoli, F. Lemoine, H. Péré, D. Veyer, J. Puech, J. Rodary, G. Baele, S. Dellicour, J. Raymenants, S. Gorissen, C. Geenen, B. Vanmechelen, T. Wawina-Bokalanga, J. Martí-Carreras, L. Cuypers, A. Sève, L. Hocqueloux, T. Prazuck, F. A. Rey, E. Simon-Loriere, T. Bruel, H. Mouquet, E. André, O. Schwartz, Considerable escape of SARS-CoV-2 Omicron to antibody neutralization. *Nature* **602**, 671–675 (2022). doi:10.1038/s41586-021-04389-z Medline

13. J. R. C. Pulliam, C. van Schalkwyk, N. Govender, A. von Gottberg, C. Cohen, M. J. Groome, J. Dushoff, K. Mlisana, H. Moultrie, SARS-CoV-2 reinfection trends in

South Africa: Analysis of routine surveillance data. medRxiv [Preprint] 2021.11.11.21266068v1 (2021); https://doi.org/10.1101/2021.11.11.21266068v1.

14. H. N. Altarawneh, H. Chemaitelly, M. R. Hasan, H. H. Ayoub, S. Qassim, S. AlMukdad, P. Coyle, H. M. Yassine, H. A. Al-Khatib, F. M. Benslimane, Z. Al-Kanaani, E. Al-Kuwari, A. Jeremijenko, A. H. Kaleeckal, A. N. Latif, R. M. Shaik, H. F. Abdul-Rahim, G. K. Nasrallah, M. G. Al-Kuwari, A. A. Butt, H. E. Al-Romaihi, M. H. Al-Thani, A. Al-Khal, R. Bertollini, P. Tang, L. J. Abu-Raddad, Protection against the Omicron variant from previous SARS-CoV-2 infection. *N. Engl. J. Med.* 10.1056/NEJMc2200133 (2022). doi:10.1056/NEJMc2200133 Medline

15. N. Ferguson, A. Ghani, A. Cori, A. Hogan, W. Hinsley, E. Volz, Imperial College COVID-19 Response Team, "Report 49: Growth, population distribution and immune escape of Omicron in England" (WHO Collaborating Centre for Infectious Disease Modelling, MRC Centre for Global Infectious Disease Analysis, Jameel Institute, Imperial College London, 2021); https://www.imperial.ac.uk/media/imperial-college/medicine/mrc-gida/2021-12-16-COVID19-Report-49.pdf.

16. UK Health Security Agency, "SARS-CoV-2 variants of concern and variants under investigation in England: Technical briefing 34" (UK Health Security Agency, 2022); https://assets.publishing.service.gov.uk/government/uploads/system/uploads/attachment_data/file/1050236/technical-briefing-34-14-january-2022.pdf.

17. E. O Murchu, P. Byrne, P. G. Carty, C. De Gascun, M. Keogan, M. O'Neill, P. Harrington, M. Ryan, Quantifying the risk of SARS-CoV-2 reinfection over time. *Rev. Med. Virol.* **32**, e2260 (2022). doi:10.1002/rmv.2260 Medline

18. C. Cohen, J. Kleynhans, A. von Gottberg, M. L. McMorrow, N. Wolter, J. N. Bhiman, J. Moyes, M. du Plessis, M. Carrim, A. Buys, N. A. Martinson, K. Kahn, S. Tollman, L. Lebina, F. Wafawanaka, J. du Toit, F. X. Gómez-Olivé, F. S. Dawood, T. Mkhencele, K. Sun, C. Viboud, for the PHIRST group, S. Tempia, SARS-CoV-2 incidence, transmission and reinfection in a rural and an urban setting: Results of the PHIRST-C cohort study, South Africa, 2020-2021. medRxiv [Preprint] 10.1101/2021.07.20.21260855 (2021); https://doi.org/10.1101/2021.07.20.21260855.

19. V. J. Hall, S. Foulkes, A. Charlett, A. Atti, E. J. M. Monk, R. Simmons, E. Wellington, M. J. Cole, A. Saei, B. Oguti, K. Munro, S. Wallace, P. D. Kirwan, M. Shrotri, A. Vusirikala, S. Rokadiya, M. Kall, M. Zambon, M. Ramsay, T. Brooks, C. S. Brown, M. A. Chand, S. Hopkins, N. Andrews, A. Atti, H. Aziz, T. Brooks, C. S. Brown, M. A. Chand, S. Hopkins; SIREN Study Group, SARS-CoV-2 infection rates of antibody-positive compared with antibody-negative health-care workers in England: A large, multicentre, prospective cohort study (SIREN). *Lancet* **397**, 1459–1469 (2021). doi:10.1016/S0140-6736(21)00675-9 Medline

20. Public Health England, "SARS-CoV-2 variants of concern and variants under investigation in England" (Public Health England, 2021); https://assets.publishing.service.gov.uk/government/uploads/system/uploads/attachment_data/file/1005517/Technical_Briefing_19.pdf.

21. P. Wang, M. S. Nair, L. Liu, S. Iketani, Y. Luo, Y. Guo, M. Wang, J. Yu, B. Zhang, P. D. Kwong, B. S. Graham, J. R. Mascola, J. Y. Chang, M. T. Yin, M. Sobieszczyk, C. A. Kyratsous, L. Shapiro, Z. Sheng, Y. Huang, D. D. Ho, Antibody resistance of SARS-CoV-2 variants B.1.351 and B.1.1.7. *Nature* **593**, 130–135 (2021). doi:10.1038/s41586-021-03398-2 Medline

22. M. Vermeulen, L. Mhlanga, W. Sykes, C. Coleman, N. Pietersen, R. Cable, R. Swanevelder, T. N. Glatt, E. Grebe, A. Welte, K. van den Berg, Prevalence of anti-SARS-CoV-2 antibodies among blood donors in South Africa during the period January-May 2021. Research Square [Preprint] (2021); https://www.researchsquare.com/article/rs-690372/v2.

23. L. Stamatatos, J. Czartoski, Y.-H. Wan, L. J. Homad, V. Rubin, H. Glantz, M. Neradilek, E. Seydoux, M. F. Jennewein, A. J. MacCamy, J. Feng, G. Mize, S. C. De Rosa, A. Finzi, M. P. Lemos, K. W. Cohen, Z. Moodie, M. J. McElrath, A. T. McGuire, mRNA vaccination boosts cross-variant neutralizing antibodies elicited by SARS-CoV-2 infection. *Science* **372**, 1413–1418 (2021). doi:10.1126/science.abg9175 Medline

24. F. Krammer, K. Srivastava, H. Alshammary, A. A. Amoako, M. H. Awawda, K. F. Beach, M. C. Bermúdez-González, D. A. Bielak, J. M. Carreño, R. L. Chernet, L. Q. Eaker, E. D. Ferreri, D. L. Floda, C. R. Gleason, J. Z. Hamburger, K. Jiang, G. Kleiner, D. Jurczyszak, J. C. Matthews, W. A. Mendez, I. Nabeel, L. C. F. Mulder, A. J. Raskin, K. T. Russo, A. T. Salimbangon, M. Saksena, A. S. Shin, G. Singh, L. A. Sominsky, D. Stadlbauer, A. Wajnberg, V. Simon, Antibody responses in seropositive persons after a single dose of SARS-CoV-2 mRNA vaccine. *N. Engl. J. Med.* **384**, 1372–1374 (2021). doi:10.1056/NEJMc2101667 Medline

25. S. Saadat, Z. Rikhtegaran Tehrani, J. Logue, M. Newman, M. B. Frieman, A. D. Harris, M. M. Sajadi, Binding and neutralization antibody titers after a single vaccine dose in health care workers previously infected With SARS-CoV-2. *JAMA* **325**, 1467–1469 (2021). doi:10.1001/jama.2021.3341 Medline

26. Y. Lustig, I. Nemet, L. Kliker, N. Zuckerman, R. Yishai, S. Alroy-Preis, E. Mendelson, M. Mandelboim, Neutralizing response against variants after SARS-CoV-2 infection and one dose of BNT162b2. *N. Engl. J. Med.* **384**, 2453–2454 (2021). doi:10.1056/NEJMc2104036 Medline

27.Department of Health – South Africa, "Update on Covid-19 (09th March 2021)" (Department of Health – South Africa, 2021); https://sacoronavirus.co.za/2021/03/09/update-on-covid-19-09th-march-2021/).

28. South African Government News Agency, "Health Department adds antigen tests to country's official COVID-19 stats" (South African Government News Agency, 2021); https://www.sanews.gov.za/south-africa/health-department-adds-antigen-tests-countrys-official-covid-19-stats).

29. A. Gelman, D. B. Rubin, Inference from iterative simulation using multiple sequences. *Stat. Sci.* **7**, 457–472 (1992). doi:10.1214/ss/1177011136

30. J. R. C. Pulliam, C. van Schalkwyk, N. Govender, A. von Gottberg, C. Cohen, M. J. Groome, J. Dushoff, K. Mlisana, H. Moultrie, Data for "Increased risk of SARS-CoV-2 reinfection associated with emergence of Omicron in South Africa," Zenodo (2022); https://doi.org/10.5281/zenodo.6108448.

31. J. R. C. Pulliam, C. van Schalkwyk, N. Govender, A. von Gottberg, C. Cohen, M. J. Groome, J. Dushoff, K. Mlisana, H. Moultrie, Code for "Increased risk of SARS-CoV-2 reinfection associated with emergence of Omicron in South Africa," Zenodo (2022); https://doi.org/10.5281/zenodo.5807591.

32. H. Sahai, A. Khurshid, *Statistics in Epidemiology: Methods, Techniques and Applications* (CRC Press, 1995).

33. K. Ulm, A simple method to calculate the confidence interval of a standardized mortality ratio (SMR). *Am. J. Epidemiol.* **131**, 373–375 (1990). doi:10.1093/oxfordjournals.aje.a115507 Medline
